# Inhibition of aldose-reductase-2 by a benzofuroxane derivative bf-5m increases the expression of kcne1, kcnq1 in high glucose cultured H9c2 cardiac cells and sudden cardiac death

**DOI:** 10.18632/oncotarget.23270

**Published:** 2017-12-14

**Authors:** Maria Consiglia Trotta, Monica Salerno, Anna Lisa Brigida, Vincenzo Monda, Antonietta Messina, Carmela Fiore, Roberto Avola, Renato Bernardini, Francesco Sessa, Gabriella Marsala, Guido N. Zanghì, Giovanni Messina, Michele D’Amico, Clara Di Filippo

**Affiliations:** ^1^ Department of Experimental Medicine, Division of Pharmacology, University of Campania L. Vanvitelli, Naples, Italy; ^2^ Department of Clinical and Experimental Medicine University of Foggia, Foggia, Italy; ^3^ Department of Experimental Medicine, Section of Human Physiology and Dietetic and Sport Medicine, University of Campania L. Vanvitelli, Naples, Italy; ^4^ Department of Biomedical and Biotecnological Sciences, University of Catania, Catania, Italy; ^5^ Struttura Complessa di Farmacia, Azienda Ospedaliero-Universitaria, Ospedali Riuniti di Foggia, Foggia, Italy; ^6^ Department of Surgery, Policlinico Vittorio Emanuele University Hospital, University of Catania, Catania, Sicily, Italy

**Keywords:** long qt interval, hyperglycemia, sudden cardiac death, BF-5m, KCNQ1 and KCNE1 ion channels, Gerotarget

## Abstract

Long QT syndrome (LQTS) is characterized by prolonged QT interval, leading to sudden cardiac death. Hyperglycemia is an important risk factor for LQTS, inhibiting the cardiac rapid component delayed rectifier K+ current (Iks), responsible for QT interval. We previously showed that the new ALR2 inhibitor BF-5m supplies cardioprotection from QT prolongation induced by high glucose concentration in the medium, reducing QT interval prolongation and preserving morphology. Here we investigated the effects of BF-5m on cell cytotoxicity and viability in H9c2 cells, and on cellular potassium ion channels expression.

H9c2 cells were grown in medium with high glucose and high glucose plus the BF-5m by assessing the cytotoxic effects and the cell survival rate. In addition, KCNE1 and KCNQ1 expression in plasma and mitochondrial membranes were monitored. Also, the expression levels of miR-1 proved to suppress KCNQ1 and KCNE1, were analyzed.

BF-5m treatment reduced the cytotoxic effects of high glucose on H9c2 cells by increasing cell survival rate and improving H9c2 morphology. Plasmatic KCNE1 and KCNQ1 expression levels were restored by BF-5m in H9c2 exposed to high glucose, down-regulating miR-1.

These results suggest that BF-5m exerts cardioprotection from high glucose in rat heart ventricle H9c2 cells exposed to high glucose.

## INTRODUCTION

Long QT syndrome (LQTS) is characterized by an abnormally long QT interval, caused by a decrease in repolarizing currents or an increase in depolarizing currents, with either congenital or acquired causes [1–2].

It is well known that in diabetic patients the onset of QT prolongation is an important complication often associated with an increased risk of sudden cardiac death due to insurgence of lethal ventricular arrhythmias known as *Torsade de Pointes* or long QT syndrome [3, 4, 5]. In this, hyperglycemia has been demonstrated to be one of the most important risk factor [6, 7], and from experimental evidence it is well known that perfusing isolated hearts with high-glucose containing Krebs solution (33mM) there is increase of QT interval and Coronary Perfusion Pressure (CPP) values in non diabetic rats [8]. *Per sè* hyperglycemia inhibits the rapid component of heart delayed rectifier K+ current (I_ks_), regulating the macromolecular complex formed by Potassium Voltage-Gated Channel Subfamily Q member 1 (KCNQ1 or Kv7.1) and Potassium Voltage-Gated Channel Subfamily E Regulatory Subunit 1 (KCNE1) [9] mainly responsible for QT interval duration in normal myocytes [10].

Further findings obtained on isolated rat hearts perfused with high-glucose Krebs solution (33 mM) showed that the new ALR2 inhibitor benzofuroxane derivative 5(6)-(benzo[*d*]thiazol-2-ylmethoxy) benzofuroxane (BF-5m) supplies cardioprotection from QT prolongation induced by the presence of a high glucose concentration into the medium [11], probably due to an interference with the potassium ion channels at the level of cardiac myocytes.

Therefore, the purpose of the study was to investigate the effects of ALR2 on cell viability and survival rate induced by high glucose in cardiac myocytes such as the embryonic rat heart ventricle H9c2 cells. Also, the expression of cellular potassium ion channels was evaluated. These cardiac myocytes provided a suitable system for our setting since they noteworthy express ALR2 receptor [12], KCNE1 and KCNQ1 subunits [13, 14]. Moreover, the expression levels of mir-1, that has been proved to suppress KCNQ1 and KCNE1 because up-regulated by hyperglycemia [15], was also evaluated.

## RESULTS

### BF-5m treatment increases the survival of the H9c2 cells following high glucose exposure

**Figure 1:**
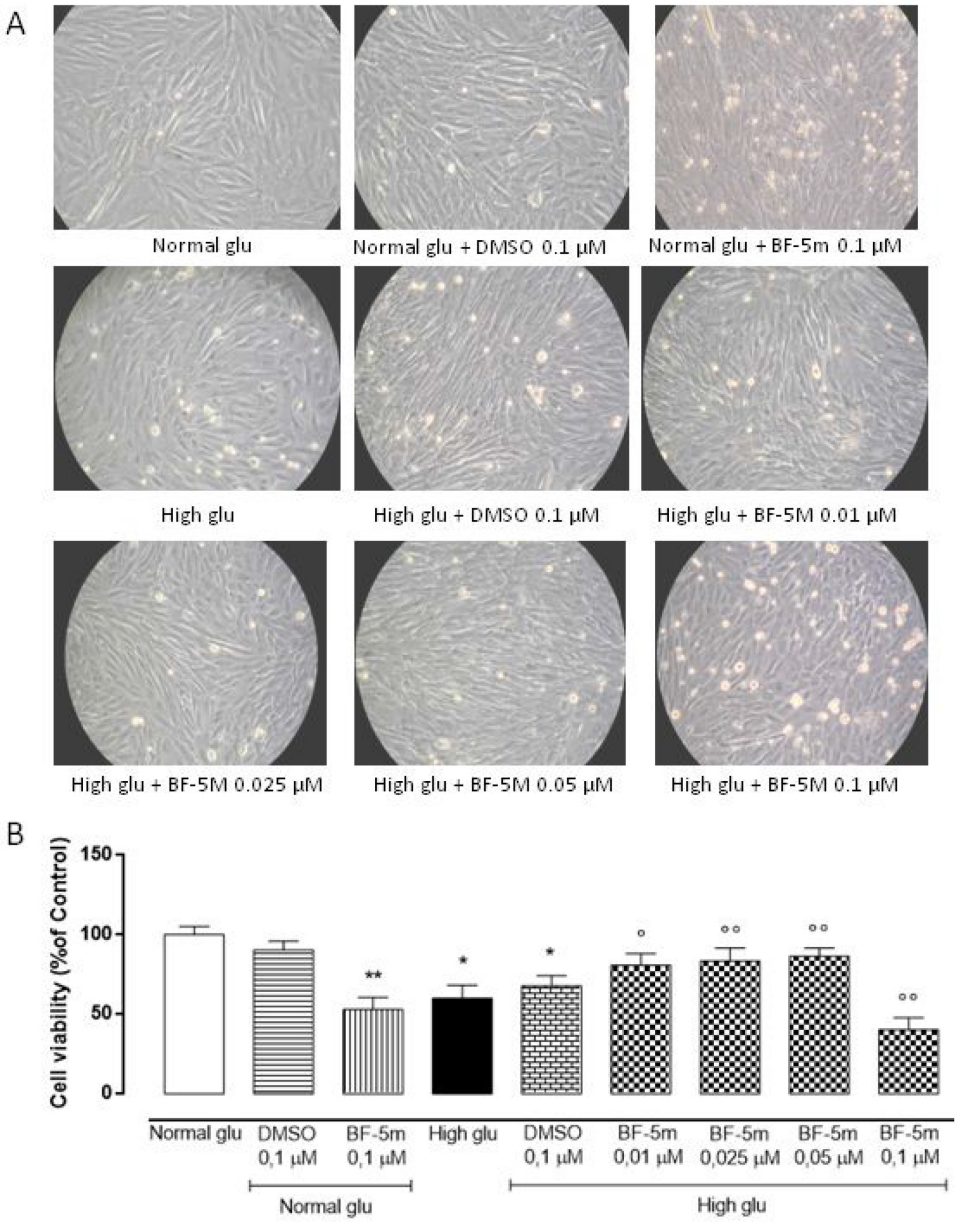
(**A**) Representative optical microscopy images at 20X magnification of H9c2 cells cultured for 24 hours in 5.5 mM glucose medium (Normal glu) and in 33 mM glucose medium (High glu) and previously exposed to DMSO 0.1 µM and BF-5m (0.01–0.025–0.05–0.1 µM). H9c2 cells pretreated with BF-5m 0.1 µM and exposed to both glucose concentrations showed an evident cell death. (**B**) MTT assay showing the cell viability as percentage of the control (Normal glu). In 5.5 mM glucose medium (Normal glu) cell survival was not affected by DMSO 0.1 µM but was significantly reduced by BF-5 m 0.1 µM. Compared to the control (Normal glu), glucose 33 mM (High glu) led to a significant decrease of the cell viability. BF-5 m 0.01–0.025–0.05 µM dose-depently increased cell survival in H9c2 exposed to glucose 33mM, while cells cultured in the same medium (High glu) and pretreated with BF-5 m 0.1 µM showed a significant reduction of cell survival. The results are reported as the mean ± S.E.M. of *n* = 3 treatments. ^*^P < 0.05 vs Normal glu; ^**^P < 0.01 vs Normal glu; °*P* < 0.05 vs High glu °°*P* < 0.01 vs High glu.

MTT assay results showed that treatment with vehicle alone (DMSO 0.1 µM) had no effects on H9c2 cells in 5.5 mM glucose medium (Normal glu) or 33 mM glucose medium (High glu). High glucose concentration (33 mM) significantly reduced cells viability compared to 5.5 mM glucose medium (*P* < 0.05 vs Normal glu). H9c2 cells exposed to high glucose (33 mM) and pretreated with BF-5 m 0.01–0.025–0.05 µM showed a significant dose-dependently increase of cell survival compared to cells exposed to high glucose alone (BF-5m 0.01 µM *P* < 0.05 vs High glu; BF-5m 0.025–0.05 µM *P* < 0.01 vs High glu), while the highest dose of BF-5m (0.1 µM) altered the amount of total cells in both glucose concentrations significantly reducing the cell viability (*P* < 0.01 vs Normal glu; *P* < 0.01 vs High glu) (Figure 1A, 1B), hence negatively interfering with mitochondrial succinate dehydrogenase activity. Therefore, the subsequent experimental settings have been performed by using doses of BF-5m lower than 0.1 µM.

### High glucose concentration induced alteration of H9c2 morphology reduced by BF-5m treatment

**Figure 2:**
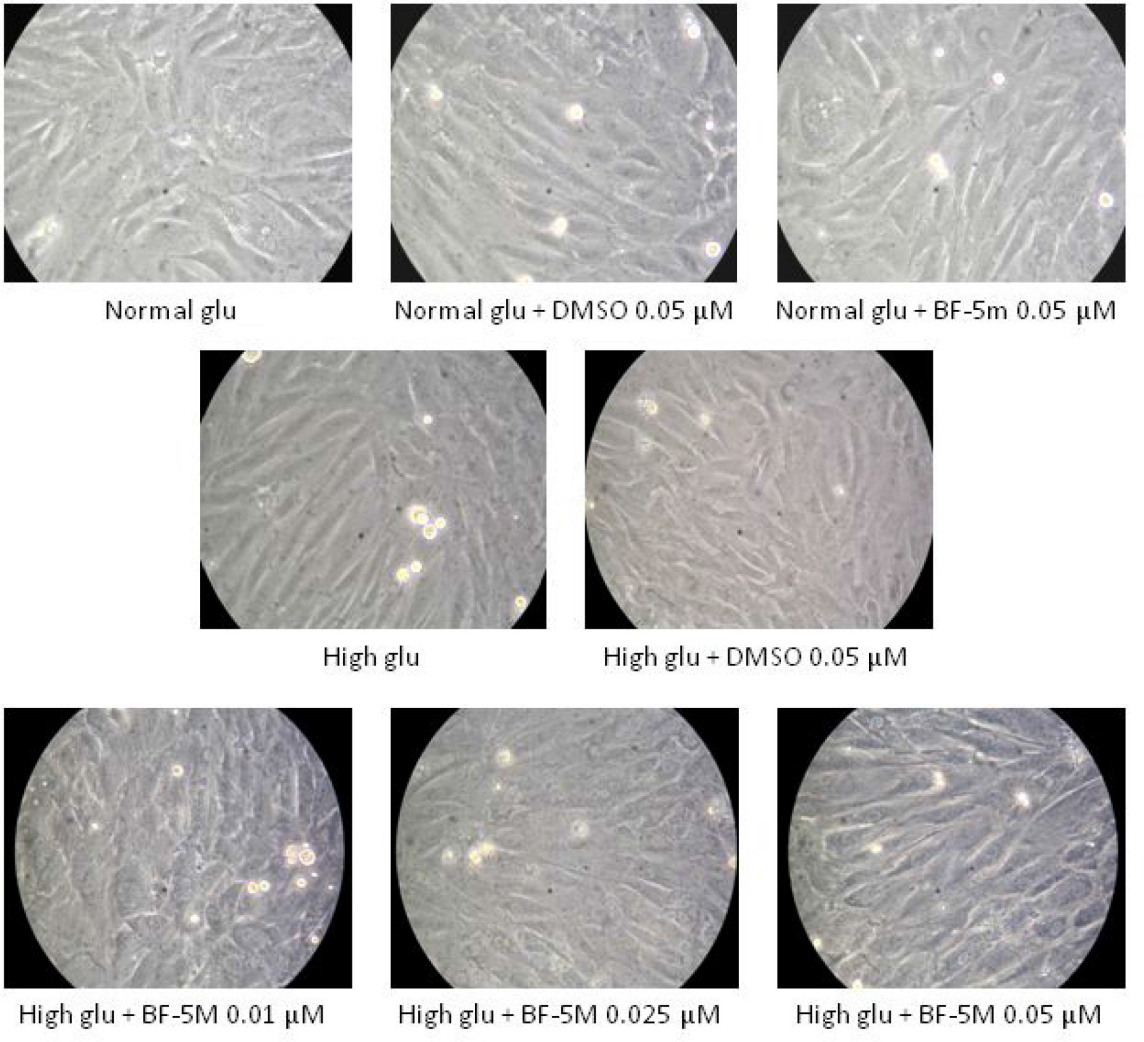
Representative optical microscopy images at 40× magnification of H9c2 cells cultured in presence of vehicle (DMSO 0.05 µM), 5.5 mM glucose (Normal glu), 33 mM glucose (High glu) and BF-5m 0.01–0.025–0.05 µM As described in test section results DMSO 0.05 µM, and BF-5 m 0.05 µM did not affect the characteristic morphology of H9c2 cells in presence of glucose 5.5 mM. H9c2 exposed to 33 mM glucose exhibited a sharply demarcated and elongated morphology compared to cells cultured in 5.5 mM glucose, while H9c2 exposed to in high glucose (33 mM) and pretreated with BF-5 m 0.01–0.025–0.05 µM showed a morphology similar to the normal one.

H9c2 cells exposed to 33 mM glucose (High glucose) exhibited an altered morphology compared to cells exposed to 5.5 mM glucose (Normal glu), being sharply demarcated and elongated (Figure 2). DMSO 0.05 µM, and BF-5m 0.05 µM did not affect the characteristic morphology of H9c2 cells in presence of normal glucose concentration (5.5 mM). H9c2 cells exposed to 33 mM and pretreated with BF-5 m 0.01–0.025–0.05 µM showed a cell shape similar to the cells grown in normal glucose medium (Figure 2).

### BF-5m pretreatment restore plasmatic KCNE1 and KCNQ1 expression levels in H9c2 exposed to high glucose

**Figure 3:**
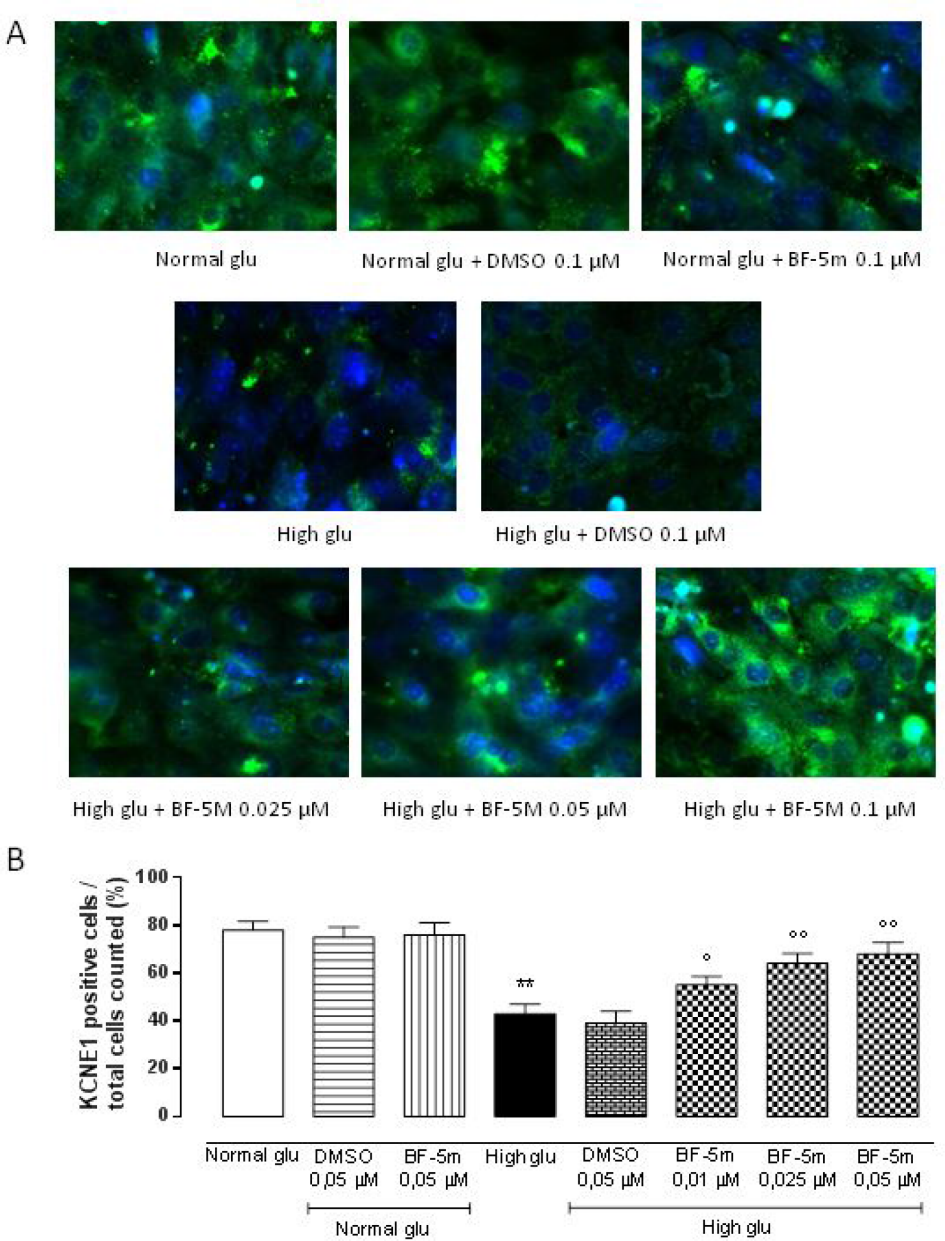
(**A**) Representative immunocytochemistries of H9c2 cells cultured in presence of glucose 11.1 mM Normal Glucosio), 33 mM glucose (High glu) and labelled with KCNE1 antibody. Representative microscopic fields for each treatment are shown with percentage of KCNE1-positive cells is represented in the graph. (**B**) The results are expressed as mean ± S.E.M. of the percentages of positive cell / total cell counted in each analyzed field for each treatment. ^**^P < 0.01 Normal glu; °*P* < 0.05 versus High glu; °°*P* < 0.01 vs High glu. 40× magnification.

**Figure 4:**
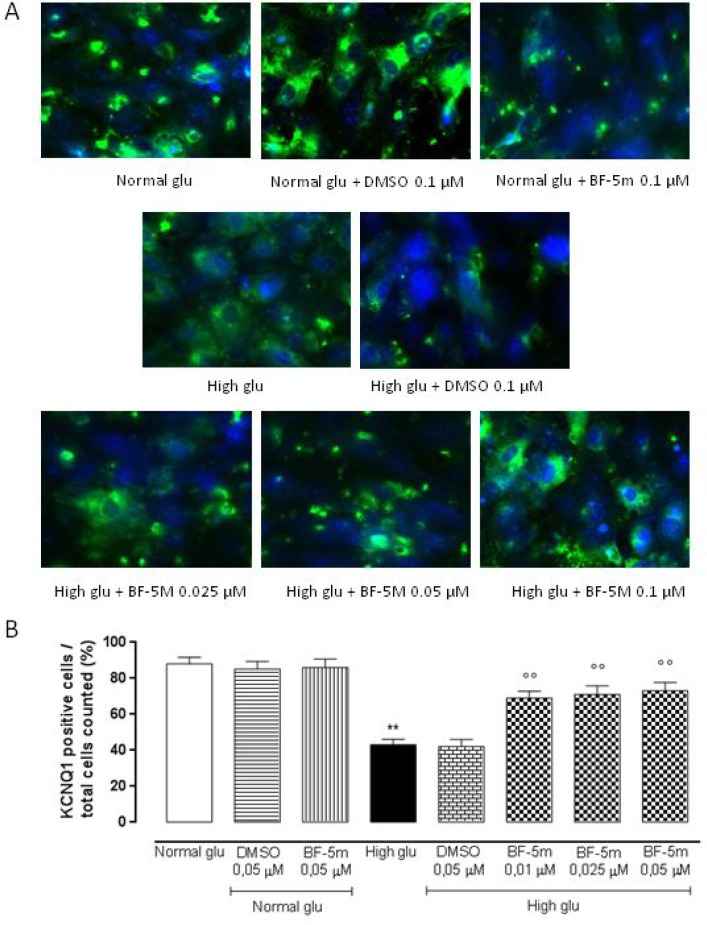
(**A**) Representative immunocytochemistries of H9c2 cells cultured in normal glucose (Normal glu) or 33 mM glucose (High glu) and labelled with KCNQ1 antibody. Cells were exposed to DMSO 0.05 µM and BF-5 m 0.01–0.025–0.05 µM. Representative microscopic fields for each treatment are shown and accordingly, the percentage of KCNQ1-positive cells is represented in the graph. (**B**) The results are expressed as mean ± S.E.M. of the percentages of positive cell / total cell counted in each analyzed field for each treatment. ^**^P < 0.01 Normal glu; °°*P* < 0.01 vs High glu. 40× magnification.

Exposure to DMSO 0.5 µM does not affect KCNE1 and KCNQ1 labeling in cells growth in normal medium or in medium supplemented with high glucose (Figures 3, 4). H9c2 cells exposed to high glucose show less KCNE1 and KCNQ1 labeling compared to cells cultured in presence of glucose 5.5 mM (*P* < 0.01 vs Normal glu) (Figures 3, 4).

In H9c2 cells cultured in presence of glucose 5.5 mM and pretreated with BF-5 m 0.5 µM there is no evidence of alterations in KCNE1 and KCNQ1 labeling (Figures 3, 4), but in H9c2 cells exposed to high glucose (33 mM), treatment with BF-5m (0.01–0.025–0.05 µM) leads to a dose-dipendently increase of the 2 subunits labeling (Figures 3, 4).

**Figure 5:**
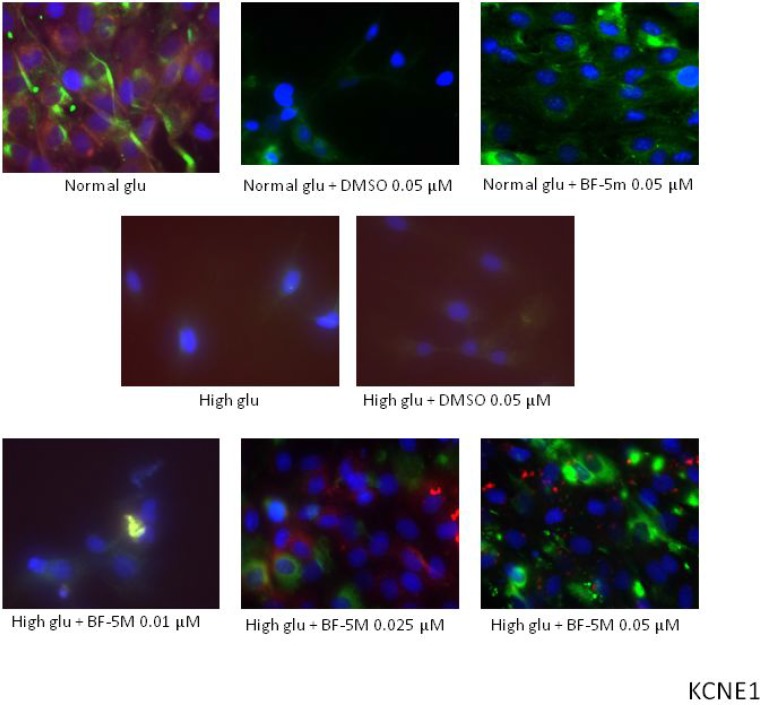
Representative merged images of MitoTracker labeling of mitochondria (red), immunofluorescence with KCNE1 antibody (green) and counterstaining of cell nuclei with Hoechst 33258 (blue) in H9c2 cells cultured in normal glucose (Normal glu) or 33 mM glucose (High glu) and exposed to DMSO 0.05 µM and BF-5m 0.01–0.025–0.05 µM Merged images did not demonstrate a significant colocalization of KCNE1 with the mitochondria in H9c2 exposed to normal or high glucose medium and receiving DMSO 0.1 µM or BF-5m 0.01–0.025–0.05 µM pretreatment. 40× magnification.

**Figure 6:**
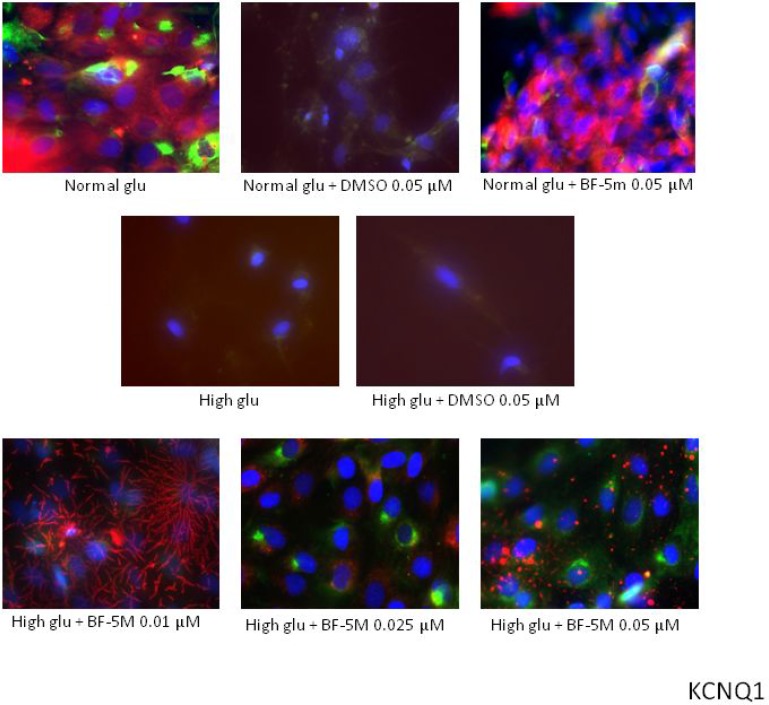
Representative merged images of MitoTracker labeling of mitochondria (red), immunofluorescence with KCNQ1 antibody (green) and counterstaining of cell nuclei with Hoechst 33258 (blue) in H9c2 cells cultured in normal glucose (Normal glu) or 33 mM glucose (High glu) and exposed to DMSO 0.05 µM and BF-5m 0.01–0.025–0.05 µM Merged images did not demonstrate a significant overlap between mitochondria and KCNQ1 in H9c2 exposed to normal or high glucose medium and receiving DMSO 0.1 µM or BF-5 m 0.01–0.025–0.05 µM pretreatment. 40× magnification.

Staining with MitoTracker^®^ Red CMXRos, showing that the mitochondrial not detected a significant co-localization of KCNE1 and KCNQ1 signals, suggesting any modification of KCNE1 and KCNQ1 mitochondrial expression levels in the different experimental conditions (Figures 5, 6). Therefore, BF-5m treatment restored the plasmatic expression of the two channel subunits.

### BF-5m pretreatment increases KCNE1 and KCNQ1 protein levels in H9c2 exposed to high glucose through a down regulation of mir-1 expression

**Figure 7:**
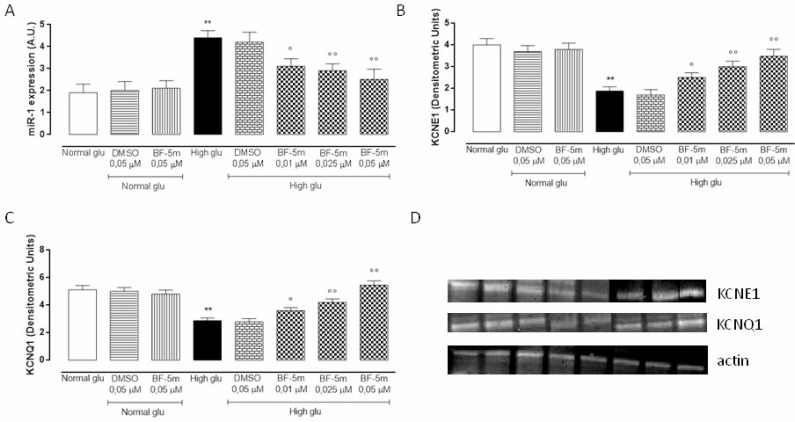
(**A**) qRT-PCR assay of miR-1 expression levels expressed as arbitrary units (A.U.) in H9c2 cultured in normal medium (Normal glu) or high glucose medium (High glu) and exposed to DMSO 0.05 µM and BF-5m 0.01–0.025–0.05 µM. H9c2 grown in high glucose medium showed an up-regulation of miR-1 levels compared to normal glu. H9c2 cultured in glucose 33 mM (High glu) and pretreated with BF-5m 0.01–0.025–0.05 µM showed a significant down-regulation of miR-1 expression levels compared to High glu. (**B**, **C**, **D**) Western blotting analysis of KCNE1 (B, D) and KCNQ1 (C, D) protein levels in H9c2 cells exposed to normal glucose (Normal glu) or high glucose (High glu) and exposed to DMSO 0.05 µM and BF-5m 0.01–0.025–0.05 µM. High glucose significantly decreased KCNE1 and KCNQ1 protein levels. Treatment with BF-5m 0.01–0.025–0.05 µM restored the KCNE1 and KCNQ1 levels. The results are reported as the mean ± S.E.M. of *n* = 3 treatments. ^**^P < 0.01 vs Normal glu, °*P* < 0.05 vs High glu and °°*P* < 0.01 vs High glu.

MiR-1 expression levels were significantly up-regulated in H9c2 cells grown in high glucose medium (*P* < 0.01) and dose-dependently down-regulated by BF-5m pretreatment (Figure 7A). In parallel, data obtained from Western Blot analysis showed a significant decrease of KCNE1 and KCNQ1 protein levels in cells exposed to 33 mM glucose compared to cells growth in normal glucose (*P* < 0.01). Pretreatment with BF-5m 0.025 and 0.05 µM significantly restored KCNE1 and KCNQ1 protein levels (Figure 7B, 7C and 7D).

## DISCUSSION

We have previously shown that the selective ALR2 inhibitor 5(6)-(benzo[𝑑]thiazol-2-ylmethoxy) benzofuroxane (BF-5m), a novel nonhydantoin noncarboxylic acid benzofuroxane derivative [18, 19], reduced QT interval in isolated rat heart perfused with Krebs solution containing high glucose concentrations [11]. In order to gain insights into the molecular targets of BF-5m we translated the previous above observation on cultured embryonic rat heart ventricle H9c2 cells under high glucose stimulus and show here that the ALR2 inhibitor BF-5m is able to increase cell viability, by reducing the cytotoxic effects induced by hyperglycemia. BF-5m increases also the expression levels of plasma membrane KCNE1 and KCNQ1 potassium ion channels and does not affect those at the mitochondrial level.

Voltage-gated potassium channels are channels that play a crucial role in returning the depolarized cell to a resting state during action potentials [20]. They, noteworthy, mediate the late repolarization phase of the action potential through two outward delayed rectifier K+ currents (IKs) [21] composed of a rapidly activating current (IKr) conducted by ether-a-go-go-related gene, hERG1 (Kv11.1; gene: KCNH2) and a slowly activating current (IKs) conducted by channels formed by coassembly of KCNQ1 (Kv7.1; gene: KCNQ1) α-subunits and auxiliary KCNE1 β-subunits [22, 23]. Disorders of functionality but also of the expression of these last channel subunits at the levels of the heart determines reduction of IKs currents and cardiac QT interval prolongation [9, 24, 25].

QT interval prolongation is considered the main cause of sudden death in diabetic patients and finds its *primum movens* in the hyperglycemia or high glucose levels into the heart as observed both in clinical and experimental settings [26]. Marfella et al. described for the first time the effect of acute hyperglycemia on QTc duration in healthy men [27]. D’Amico et al. in 2001 demonstrated that high glucose levels into the heart induce ventricular instability characterized by QT interval prolongation [8], an evidence subsequently continued by Di Filippo et al., in 2002 [28]. More appropriately, Morales-Cano et al., found that hyperglycemia down-regulates KV7 potassium channels at the level of coronary artery influencing their reactivity [29]. The same author in 2016 confirmed that hyperglycemia induces impairment of Kv7 channels and cAMP-mediated relaxation in rat coronary arteries [30]. All these studies, therefore, paved the way to a direct and key role of hyperglycemia/high glucose in potassium channels-linked QT interval prolongation.

Hyperglycemia/high glucose, however, causes alterations of factors that may be indirect actors in biomolecular and electrical changes induced by the condition of high glucose into the heart. Among these is the hyperactivity of the enzyme ALR2, the first and rate-limiting enzyme of the polyol pathway, that catalyzes the reduction of glucose to sorbitol through the oxidation of NADPH to NADP [19]. Hyperactivity of this enzyme increases the levels of reactive oxygen species (ROS) [31], which noteworthy affect membrane potassium ion channels activity [32, 33] leading cardiac QT prolongation at the level of the heart [34].

On this base the initial inhibition of the endogenous ALR2 with a benzofuroxane derivative BF-5m [11] represented a new and important strategy to indirectly approach high glucose-induced QT interval prolongation. Insights acquired here show for the first time that the blockade of the ALR2 with BF-5m causes changes of the expression of H9c2 KCNQ1/KCNE1 potassium channels subunits at the plasma membrane level and not at the level of the mitochondria after high glucose exposure. Interestingly, MitoTracker assay showed a mitochondrial KCNE1 and KCNQ1 expression, not affected by high glucose compared to normal glucose concentration. This is quite surprising because one would have expected hyperglycemia to alter these mitochondrial channels and no others. Evidence exists, in fact, on alterations of mitochondrial respiratory chain complex associated to prolongation of cardiac QT interval and hyperglycemia [2, 5, 34] as well as MTT assay based on mitochondrial succinate dehydrogenase activity, part of mitochondrial complex II, showed reduced cell viability from high glucose exposure. On another note, BF-5m per sè did not lead to any changes of the two potassium channel subunits in H9c2 cells exposed to normal or high glucose conditions. One would have expected that under high glucose stimulus the activation of protein kinase C (PKC) isoforms would have induced phosphorylation of ALR2 and its translocation to the mitochondria [35, 36], where ALR2 contributes to high glucose-mediated mitochondrial dysfunction and, perhaps, ion channels alterations through the phosphorylation and activation of p53 [37]. Therefore, the plasma membrane KCNQ1/KCNE1 are the one modified by high glucose and ALR2 inhibition in H9c2 cells. In line with these concepts is the evidence that all functional roles of Kv7.1 channels (KCNQ1/KCNE1) have been previously attributed to their plasma location while no such role is attached to the mitochondrial one, at least for what concerns the cardiac control. Testai et al. (2016) (25) proved recently that KCNQ4 (or Kv7.4) channels are the one activate in rat cardiac mitochondria exerting a significant cardio-protective role against ischemia/reperfusion-induced cardiac injury.

From the epigenetic point of view, the expression of the KCN- potassium channels macromolecular complex is targeted and down-regulated by abnormal expression of miR-1, caused by hyperglycemia stimulation [13]. This miRNA has been found to provoke cardiac arrhythmias by altering potassium current in mature myocytes [38]. Effectively, in the present study immunocytochemistry and western blot analysis showed that H9c2 cells exposed to high glucose have low levels of KCNQ1/KCNE1 and high levels of miR-1. BF-5m dose-dependently increased plasmatic KCNE1 and KCNQ1 levels, significantly reduced by high glucose medium, paralleled by a significant down-regulation of miR-1 levels. This latter evidence being a further novelty of this study since no one have previously linked ALR2 to miR-1 or KCN- potassium channels.

Finally, morphological analysis of H9c2 cells by optical microscope showed that the selective inhibition of ALR2 with BF-5m improved the sharply demarcated and stretched elongated morphology characteristic of cardiomyocytes grown in hyperglycemic conditions for 24 hours. This, together with cell viability, indicates a conservation of cell biology after BF-5m and may further support a possible prevention from sudden cardiac death through a putative preservation of the normal electrical activity of the myocytes induced by the compound. In fact, the reduction of mortality from sudden cardiac arrest in the setting of coronary heart disease remains a major challenge, especially for patients with type 2 diabetes [39]. It is well described that patients with higher baseline blood glucose levels have a significantly increased risk of heart failure [40].

In conclusion, these results suggest that the new aldose reductase inhibitor benzofuroxane derivative BF-5m may supply cardioprotection from the high glucose induced instability of QT interval components by reducing the cytotoxic effects induced by hyperglycemia on cell viability, by down-regulating miR-1 expression and consequently restoring plasma membrane KCNE1 and KCNQ1 levels in rat heart ventricle H9c2 cells exposed to high glucose.

## MATERIALS AND METHODS

### Drug

BF-5m, 5(6)-(benzo[*d*]thiazol-2-ylmethoxy) benzofuroxane, was synthesized at the Department of Pharmacy of the University of Pisa, Italy, as previously reported [11] and dissolved in DMSO 1% (CAS 67–68–5 Fisher Scientific, Italy).

### H9c2 cell culture

H9c2 (2-1) cardiomyocytes (88092904 Sigma, Italy) were grown in Dulbecco’s modified Eagle’s medium (DMEM) (AU-L0101-500 Aurogene, Italy) containing 5.5 mM glucose [16] (A24940-01, Thermo fisher, Italy, supplemented with 10% fetal bovine serum Heat Inactivated (AU-S181H-500 Aurogene, Italy), 5% L-Glutamine (AU-X0550-100, Aurogene, Italy) and 5% Penicillin-Streptomycin Solution (AU-L0022-100, Aurogene, Italy), at 37°C under an atmosphere of 5% CO2. At a density of 50 × 10^5^ cell/mL, BF-5m (0.01–0.025–0.05–0.1 µM) or vehicle DMSO (0.05 –0.1 µM) was added to H9c2 cells. After 1 day of treatment, they were exposed to 33 mM glucose (A24940-01, Thermo fisher, Italy) [17] for 24 hours. Each treatment was repeated three times and H9c2 cell morphology was daily observed with optic microscope (Leica DMi1, Germany).

### Cell viability assay

Cell viability was measured by 3-(4,5-dimethylthiazol-2-yl)-2,5-diphenyltetrazolium bromide assay (MTT). Cells (5 × 10^3^ cells/well) were seeded in 96-well plates and exposed to vehicle DMSO (0.05–0.1 µM) and BF-5m (0.01- 0.025–0.05–0.1 µM) for 24 hours. Then, glucose solution was added to growth medium to reach a final glucose concentration of 33 mM. After 24 hours the MTT solution (1:10 in culture medium) was added to each well of 96-well plates. The cells were cultured for 4 hours and then washed for 20 min in isopropanol-HCl 0,2 N, following the manufacturer’s instructions. Optical density (OD) values were measured at 570 nm using a 96-well plate reader (iMark, Bio-Rad Laboratories, Italy).

### Immunocytochemistry

For immunocytochemical analysis, H9c2 were resuspended at 50 × 10^4^ cell/mL in DMEM medium containing 10% FBS, 5% L-glutamine, 5% Penicillin-Streptomycin Solution (all Aurogene, Italy), plated on slides with a 24-well plate and incubated at 37°C with 5% CO_2_. To detect mitochondrial expression of KCNE1 and KCNQ1, cells were incubated with MitoTracker^®^ Red CMXRos (M7512 ThermoFisher Scientific, Italy) before fixation with 4% paraformaldehyde.

After washing in PBS, non-specific antibody binding was inhibited by incubation for 30 minutes in blocking solution (1% BSA in PBS). Primary antibodies were diluted in PBS blocking buffer and slides were incubated overnight at 4°C in primary antibodies to rat KCNE1 (1:50; MBS8503082, My BioSource, USA) or to rat KCNQ1 (1:200; bs-6760R Bioss Inc., USA). Fluorescent-labeled anti-rabbit secondary antibody (1:1,000; Alexa Fluor 488, Molecular Probe; Invitrogen, Carlsbad, CA, USA) was used to locate the specific antigens in each slide. Cells were counterstained with bisbenzimide (Hoechst 33258; Hoechst, Frankfurt, Germany) and mounted with mounting medium (90% glycerol in PBS). Fluorescently-labeled slides were viewed with a fluorescence microscope (Leica, Wetzlar, Germany) and with a fluorescence confocal microscope (LSM 710, Zeiss, Oberkochen, Germany). Immunofluorescence images were analyzed with Leica FW4000 software (Leica, Wetzlar, Germany) and with Zen Zeiss software (Zeiss, Oberkochen, Germany). Quantification was performed by an observer blind to the treatment. The percentage of positive cells in each microscope field was calculated by the number of green positive cells of 300 cells in four different microscope fields. Only bisbenzimide counterstained cells were considered as positive profiles so as to avoid overcounting cells. Cell positive profile quantification was performed on each digitized image, and the reported data are the intensity means ± SEM on counterstained cells per group for each treatment, repeated three times.

### RNA extraction and mir-1 expression

Total RNA isolation was performed using the miRNeasy Mini kit (Qiagen, Italy) according to the supplementary protocol Purification of Total RNA, including Small RNAs, from Animal Cells. In order to monitor the efficiency of miRNA recovery and to normalize miRNA expression in the Real-time PCR experiment, a 5 μL aliquot of 5 nM Syn-cel-miRNA-39 miScript miRNA Mimic was spiked into each sample before nucleic acid preparation. RNA was then quantized using NanoDrop 2000c Spectrophotometer (Thermo Fisher Scientific, Waltham, MA USA). Total RNA was then reverse-transcribed using the miScript II RT kit (SABiosciences) according to manufacturer’s protocol. For a single PCR reaction we used 12.5 μl of 2× QuantiTect SYBR Green PCR Master Mix (QIAGEN), 2.5 μl of template cDNA (10 times diluted), 2.5 μl of 10x miScript Primer Assays (QIAGEN), containing a primer for a specific miRNA (miR-1 and Syn-cel-miR-39-3p), and RNAse free water (QIAGEN) to a final volume of 25 μl. Each reaction was carried out in triplicate in Bio-rad CFX96 cycler (Bio-Rad Laboratories, Inc). Relative quantization of gene expression was performed calculating the DCt value for each miRNA as Ct miRNA–Ct Syn-cel-miR-39; expression fold change was then obtained as 2^-DCt. The *P*-values are calculated based on a Student’s *t*-test of the replicate 2^-DCt values for each miRNA in the control and pre-treated groups. *P* < 0.05 was considered significant.

### Western blot assay

At the end of the treatments, H9c2 cells were harvested and lysed in RIPA buffer (R0278 Sigma, Italy). The homogenate was centrifuged at 12,000 rpm for 10 min at 4°C and the protein concentrations in the supernatant were quantified with Bio-Rad protein assay (500-0006 Bio-Rad Laboratories, Italy). After the gel electrophoresis in a 10% PAGE separation gel, 20 μg of protein sample were electro transferred onto a PVDF membrane. Blots were blocked with 5% non-fat dry milk for 1h at room temperature, and then incubated with primary specific antibodies over-night, followed by incubation with a horseradish peroxidase-conjugated secondary antibody for 1 h at room temperature. The signal was expressed as densitometric units (DU).Western blots were performed to evaluate the expression levels of KCNE1 and KCNQ1 using of the following primary antibodies: anti-KCNE1 (1:1000 MBS8503082 My BioSource, USA) anti-KCNQ1 (1:1000 ab192425 abcam, UK) and anti-actin (1:500 a3853 Sigma, Italy). For all assays, goat anti-rabbit (1:5000 ADI-SAB-300) and anti-mouse (1:5000 ADI-SAB-110 Enzo, Italy) HRP horseradish peroxidase were used as secondary antibodies.

### Statistical analysis

The results of each experiment are presented as mean ± S.E.M. of the three treatments. Statistical significance was determined using ANOVA followed by Bonferroni’s test. A probability *P* value less than 0.05 was considered significant to reject the null hypothesis.
